# Future of Sclerotherapy in the Treatment of Endometriosis: A Narrative Literature Review

**DOI:** 10.7759/cureus.81215

**Published:** 2025-03-26

**Authors:** Dimitrios Lentzaris, Fani Gkrozou, Chara Skentou, Nikoleta Koutalia, Vasileios Bais, Anastasia Vatopoulou, Minas Paschopoulos

**Affiliations:** 1 Obstetrics and Gynaecology, University Hospital of Ioannina, Ioannina, GRC

**Keywords:** cystectomy, endometriomas, endometriosis, fertility preservation, ovarian cysts, ovarian reserve, sclerotherapy

## Abstract

Endometriosis is a gynaecological condition affecting reproductive-age women. In case of ovarian endometriosis, there is cystic formation, called endometrioma. Although laparoscopic cystectomy is established as the gold standard for the treatment of endometriomas, it is related to damage of healthy ovarian tissue and significant decrease in ovarian reserve. As a result, new strategies have been set for the care of endometriomas, with ethanol sclerotherapy as the most popular alternative. As a part of sclerotherapy, ethanol solution is injected to destroy pseudocapsule. Iatrogenic damage is minimized, making it a safe and less invasive technique. In this study, we compared ultrasound-guided (U/S) ethanol sclerotherapy and laparoscopic ethanol sclerotherapy with laparoscopic cystectomy. Anti-Müllerian hormone (AMH) is used to monitor ovarian preservation. Sclerotherapy seems to have smaller effect on ovarian reserve compared to cystectomy. Recurrence rate and clinical pregnancy rate are similar, though cystectomy has better outcome of symptom relief. Moreover, laparoscopic sclerotherapy seems to achieve the best clinical pregnancy rate and decrease recurrence rate.

## Introduction and background

Endometriosis is an estrogen-dependent gynaecological condition, where functional endometrial tissue appears outside the uterus [1]. This results in the development of chronic inflammation and fibrous connective tissue in the pelvis or anywhere else in the body [2]. The most common locations of the disease are the ovaries, fallopian tubes, pelvic peritoneum and uterosacral ligaments, while the gastrointestinal tract, the urinary system, soft tissues, and chest are more rare [3].

Pathophysiology of endometriosis is not completely understood [4]. Three main mechanisms have been proposed. The retrograde menstruation, the reverse flow of blood full of endometrial cells during menstruation towards the fallopian tubes and peritoneal cavity, resulting in the implantation of endometrial cells outside the uterus and their subsequent proliferation [3]. Cellular metaplasia theory refers to the transformation of cells outside the uterus into endometrial-like cells and their further development. Finally, the third theory is the manifestation of the disease by stem cells, which are then dispersed through the blood and lymphatic pathways [5].

Endometriosis is a common gynaecological condition with overall prevalence of 18% [6]. Signs and symptoms of the disease are related to its location and include chronic pelvic pain, pain associated with menstruation (dysmenorrhoea), pain during intercourse (dyspareunia), periodic or menstrual-related gastrointestinal and urinary symptoms, as well as infertility [7]. In fact, endometriosis is a main cause of secondary dysmenorrhoea, especially in young women, with a serious impact on various aspects of everyday life. Τhis results in limitations in daily activities, as well as adverse effects on academic performance and sleep quality. It also has a negative psychological impact, intensifying anxiety and leading to depression [8]. Regarding endometriosis and infertility, there is a strong correlation, since about 30 to 50% of women with endometriosis have infertility, while 25 to 50% of infertile women have endometriosis [9]. Several mechanisms have been proposed, including peritoneal dysfunction and distorted pelvic anatomy, and even altered endocrine and endometrial cell-mediated functions [9].

The diagnosis of endometriosis is definitively established when the presence of endometrial tissue outside the uterus is confirmed during surgery, typically by laparoscopic approach. Αs far as non-invasive diagnostic methods are concerned, they include transvaginal ultrasonography and MRI, which can be used for the detection of ovarian and deep endometriosis as well. Indeed, the advance in diagnostic techniques using MRI in recent years has significantly increased the preoperative diagnosis of deep endometriosis [10]. Endometriotic foci can also be observed in asymptomatic patients. Data from epidemiological studies indicate that women with endometriotic lesions have a higher risk of developing ovarian and breast cancer, melanoma, asthma, and rheumatoid arthritis, as well as developing diseases of the cardiovascular system [11].

In case of ovarian endometriosis, there is cystic formation, called endometriomas or chocolate cysts due to the dark appearance of their contained fluid [12]. About 17-44% of women diagnosed with endometriosis will develop endometriomas [13].

Treatment of endometriosis can be conservative or surgical [14]. Conservative management includes combined oral contraceptive pills, oral progestogens, implants contain progestogen or progesterone releasing intrauterine device [15]. The main concern regarding the pharmaceutical treatment of endometriosis is the control of pain, the prevention of recurrences, the improvement in the quality of life as well as the preservation of fertility. Endometriosis-related pain is due to the inflammatory environment associated with the disease, in the pathogenesis of which estrogen plays a key role. Effective treatments for management of endometriosis-related pain, therefore, focus on suppressing ovulation and estrogen production. In this way, hormone therapy inhibits inflammatory processes and prevents the progression of the disease by creating a relatively hypoestrogenic environment. However, its action only lasts for the duration of the treatment, which results in the reappearance of the pain and other endometriosis-related symptoms after its discontinuation [16]. Furthermore, some cases require more aggressive approach. In deep endometriosis, it is deep endometriosis resection with adhesion stripping laparoscopically, and with respect to endometriomas, it is ovarian cystectomy performed by laparoscopy [17]. In these cases, as endometriomas are surrounded by a pseudocapsule, healthy ovarian tissue is inevitably removed during cystectomy. In addition, the use of energy in order to achieve haemostasis leads to further reduction in the ovarian reserve [18].

During sclerotherapy, the effect on ovarian tissue is different. More specifically, the endometriotic fluid is removed by suction, its cavity is washed out and a solution of ethanol is injected to destroy the pseudocapsule [19]. In this way, iatrogenic damage is possibly minimized, ovarian parenchymal preservation optimized, and sclerotherapy standardized as a safe and less invasive technique [20].

The present study aims to compare the two available options of ethanol sclerotherapy, ultrasound-guided (U/S) and laparoscopic sclerotherapy, with laparoscopic cystectomy, which is the gold standard treatment. The comparison took into account the effect on the ovarian reserve, monitored by anti-Müllerian hormone (AMH), the effect on cancer antigen 125 (Ca-125), recurrence rate, symptom relief, clinical pregnancy rate, and finally the live birth rate after assisted reproductive technology (ART).

## Review

Materials and methods

We performed research in PubMed, EMBASE, Scopus, Clinical-trials, and Google Scholar on studies comparing the results of sclerotherapy for endometriomas through laparoscopy or ultrasound guidance with laparoscopic cystectomy for the last 10 years. Studies only in English language were included. We used the following string of idioms in each database to identify studies fitting to our review’s topic: “sclerotherapy for endometriosis”, “laparoscopic cystectomy and endometriomas”, “ultrasound guided sclerotherapy for endometriomas”, “laparoscopic sclerotherapy for endometriomas”.

Study selection was made by F.G. and D.L. The inclusion criteria are below: studies including patients with at least one ovarian endometrioma and treated with sclerotherapy or cystectomy. We included the studies that reported at least one outcome of interest regarding effect on ovarian reserve (using AMH), effect on Ca-125, recurrence rate, symptom relief, clinical pregnancy rate after the procedure, live birth rate after ART. Our study includes only published peer-reviewed original articles. We excluded non-original studies, pre-clinical trials, animal trials, meta-analysis, abstract-only publications, and articles in a language other than English. In the last 10 years, 36 studies were published in English language, though only nine met our inclusion criteria. Table 1 summarizes the studies selected, with details such as year of publication, study design, number of participants, and followed procedure.

**Table 1:** Studies included [21-29] Lap: Laproscopic; U/S: Ultrasound-guided

Authors	Year of publication	Study type	Number of patients (N)	Procedure
Alborzi et al.	2021	Prospective cross-sectional study	101	Lap cystectomy vs U/S sclerotherapy
Koo et al.	2021	Retrospective study	71	Lap cystectomy vs U/S sclerotherapy
Miquel et al.	2020	Retrospective cohort study	74	U/S sclerotherapy vs no intervention
Garcia-Tejedor et al.	2020	Prospective multi-center cohort study	31	Lap cystectomy vs U/S sclerotherapy
Ghasemi Tehrani et al.	2022	Randomized clinical trial	70	Lap cystectomy vs U/S sclerotherapy
De Cicco Nardone et al.	2020	Retrospective study	53	Lap sclerotherapy
Vaduva et al.	2023	Retrospective study	96	Lap cystectomy vs U/S sclerotherapy
Lee et al.	2022	Retrospective study	18	U/S sclerotherapy
Crestani et al.	2023	Observational study	69	Lap sclerotherapy

Studies' characteristics

**Figure 1:**
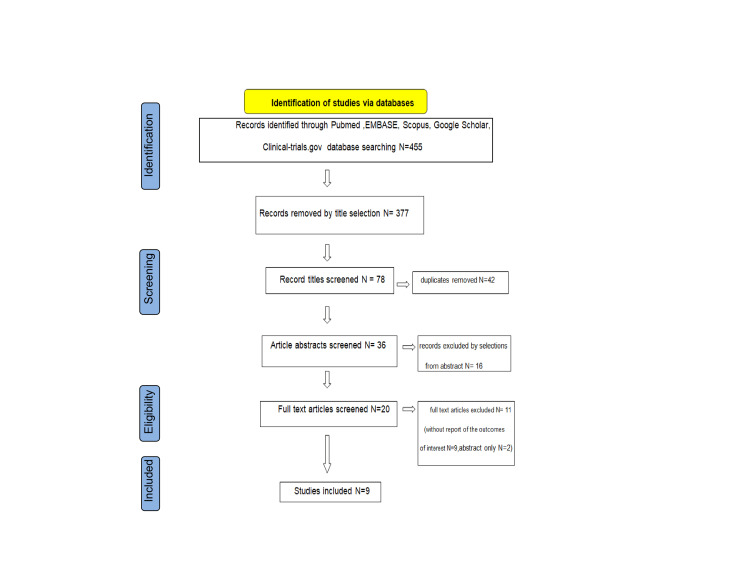
Study selection PRISMA flow diagram PRISMA: Preferred Reporting Items for Systematic Reviews and Meta-Analyses

After searching databases, 455 results were identified. From them, 377 were excluded by title selection, while 42 were duplicates. From the 36 articles remaining, 20 were included after abstract screening. A total of nine studies were excluded after full text reading since they didn’t report any of the outcomes of interest (effect on ovarian reserve using AMH, effect on Ca-125, recurrence rate, symptom relief, clinical pregnancy rate after the procedure, or live birth rate after ART) [20,30-37]. Two more were excluded as abstract-only articles [38,39]. We mentioned the studies selected and all reasons for exclusion in the Preferred Reporting Items for Systematic Reviews and Meta-Analyses (PRISMA) flowchart (Figure 1).

Results

A total of 583 patients were included in this review [21-29]. The studies publication years range from 2020 to 2023 while the follow-up period ranged from 3 to12 months. Ovarian reserve is measured by using AMH. Information about mean level of AMH was given only for a total of 144 patients that had U/S sclerotherapy [22,24,25,27,28]. For this group, mean AMH level was found to be 1.37 ng/ml before U/S sclerotherapy, while the result after the procedure was 1.34 ng/ml, noting a 2.18% reduction of AMH. When laparoscopic sclerotherapy is studied, information was given only for 69 patients [29]. Preoperative mean AMH levels were 1.92 ng/ml, while postoperative mean levels were 1.13 ng/ml, noting a reduction of 41.14%. Finally, for the 142 patients who underwent laparoscopic cystectomy and had their AMH levels measured, the mean AMH level before the procedure was 1.82 ng/ml, and mean level after the procedure was 1.05 ng/ml, having an AMH reduction percentage of 42.30% [22,24,25,27].

In a total of 225 patients who underwent U/S sclerotherapy, 77 (34.22%) had their symptoms improved or resolved [22,24,25,27,28]. From a total of 199 patients who underwent laparoscopic cystectomy, 78 patients (39.19%) had their symptoms improved or resolved [22,24,25]. No data was noted for the group of laparoscopy sclerotherapy and symptoms improvement.

Endometrioma recurrence was noted at 38 out of 225 patients that underwent U/S sclerotherapy, with a total recurrence rate of 16.88% [21,22,24,25,27,28]. In the group of patients who underwent laparoscopic sclerotherapy, 11 had recurrence of endometriomas, having a total recurrence rate of 9.01% [27,28]. This is in contrast with the patients in the laparoscopic cystectomy group, for whom the recurrence rate went up to 15.57% [21,22,24,25].

Among 225 patients who underwent U/S sclerotherapy, 32 achieved a clinical pregnancy (14.22%) [21,23,24]. 34 out of 122 patients that underwent laparoscopic sclerotherapy achieved clinical pregnancy (27.87%) [27,28]. Finally, from a total of 199 patients who had a laparoscopic cystectomy, 24 achieved clinical pregnancy, with a total clinical pregnancy rate of 12.06% [21,24].

A live birth rate of 11.11% was achieved after ART in women who had U/S sclerotherapy [21,23]. Furthermore, 22 of the 199 patients who underwent laparoscopic cystectomy gave birth with a live birth after ART, a rate of 11.05% [21]. No data is available for the laparoscopic sclerotherapy group.

Ca-125 levels were measured before and after U/S sclerotherapy in 38 patients, with initial mean Ca-125 level of 11.77 U/ml, and mean level after the procedure being 4.74 U/ml, noting a reduction of 59.73% [31,32]. There was no data about Ca-125 in the laparoscopic sclerotherapy group. Finally, 51 patients who underwent laparoscopic cystectomy had their Ca-125 levels measured pre and post operatively, with preoperative mean level 7.36 U/ml and post operative one 2.25 U/ml, with a reduction of 69.43% [22].

The results mentioned above are summarized in Table 2.

**Table 2:** Results AMH: Anti-Müllerian hormone; U/S: Ultrasound-guided; ART: Assisted reproductive technology

Parameters	U/S Sclerotherapy	Lap Sclerotherapy	Lap Cystectomy	References
	N = 225	N = 122	N = 199	
AMH (ng/mL)	Mean AMH before = 1.37ng/ml	Mean AMH before = 1.92ng/ml	Mean AMH before = 1.82ng/ml	[32,34-37,39]
	Mean AMH after = 1.34ng/ml	Mean AMH after= 1.13ng/ml	Mean AMH after = 1.05ng/ml	
					Reduction percentage = 2.18%	Reduction percentage = 41.14%	Reduction percentage = 42.30%
	Quality of Life	Symptoms resolved or improved = 77	-					Symptoms resolved or improved = 78	[34-37,39]
										34.22% of patients	39.19% of patients
		Recurrence						Recurrence = 38				Recurrence = 11	Recurrence = 31	[31-37,39]
					Recurrence percentage = 16.88%	Recurrence percentage = 9.01%	Recurrence percentage = 15.57%							
Clinical Pregnancy Rate	Clinical pregnancy = 32		Clinical pregnancy = 34	Clinical pregnancy = 24				[31-34,38]						
	Clinical pregnancy rate = 14.22%		Clinical pregnancy rate = 27.87%	Clinical pregnancy rate = 12.06%										
									Live Birth Rate after ART	Live births after ART = 25	-	Live births after ART = 22	[31,38]	
Live birth rate after ART = 11.11%	Live birth rate after ART = 11.05%													
		Ca-125 (U/mL)	Mean Ca-125 before = 11.77U/ml	-	Mean Ca-125 before = 7.36U/ml	[36,37]							
			Mean Ca-125 after = 4.74U/ml		Mean Ca-125 after = 2.25U/ml								
							Reduction percentage = 59.73%	Reduction percentage = 69.43%					

Discussion

This study aims to present results after comparing laparoscopic cystectomy, U/S sclerotherapy, and laparoscopic sclerotherapy in the treatment of endometriomas.

Although endometriosis is a common disease and there is a high prevalence of endometriomas, there is still no definitive treatment that will result in a cure without any cost to the ovarian reserve. Endometrioma has a pseudocyst; consequently, the risk of removing normal ovarian tissue during surgery is high. The gold-standard treatment, laparoscopic cystectomy, has been found to reduce the ovarian reserve with inevitable effect on achieving pregnancy. Busacca et al. have presented three cases of patients aged 31, 33, and 39 years with premature ovarian failure right after the laparoscopic excision of bilateral endometriomas, even though they all had regular menstruation before surgery [40].

U/S sclerotherapy caused 2.18% reduction in AMH levels, while laparoscopic sclerotherapy and laparoscopic cystectomy caused 41.14% and 42.30% reduction, respectively. These findings are consistent with a study by Raffi et al., which reported 38% decline in AMH levels after cystectomy, as well with the meta-analysis by Kim et al. where there was no significant difference in AMH level before and after U/S sclerotherapy [41,42].

Although laparoscopic sclerotherapy seems to cause similar reduction in AMH levels as laparoscopic cystectomy, we believe that is due to the small number of patients for the laparoscopic sclerotherapy group. Moreover some studies did not mention the mean follow-up time, making it hard to extract reliable results. Finally, it is known that short-term postoperative AMH levels are lower due to ovarian recovery after surgery [43].

Regarding symptoms caused by endometriosis, 34.22% of patients that underwent sclerotherapy with ultrasound had their symptoms resolved or improved, while 39.19% of patients after laparoscopic cystectomy managed to have symptom relief, something that shows that cystectomy can be more efficient at that matter but still it is no very well studied its results in future pregnancy ability.

When clinical pregnancy rate was assessed, data indicated 14.22% and 12.06%, respectively, for U/S sclerotherapy and laparoscopic cystectomy, while laparoscopic sclerotherapy group noted a clinical pregnancy rate of 27.87%. This is quite impressive that indicates possible the superiority of laparoscopic sclerotherapy. In that case, ovarian tissue is preserved better and also at the same time any other area of endometriosis can be excised. As result the inflammatory background is limited fact that potentially contributes to the increase of clinical pregnancy [44].

Laparoscopic cystectomy is related with a 69.43% reduction of Ca-125, while in the group of U/S sclerotherapy, Ca-125 was reduced by 59.73%, which is consistent with the findings of Han et al. [45]. Ca-125 is known to be increased in cases of endometriosis, with a sensitivity of 40% [46]. Mainly in the past, Ca-125 was used widely to detect the disease and monitor its progress. That is the reason why many studies used it as a marker to check the efficacy of different methods [40,41]. However, its use is controversial and it can be related with other pathologies [46].

Although laparoscopic cystectomy is currently the gold standard for the treatment of endometriomas, this approach is controversial when it comes to patients who undergo ARTs [47]. The reason for the controversy is the loss of healthy ovarian tissue and the reduction in ovarian reserve after such an operation. On the other hand, endometriomas have a negative effect on fertility as their presence is related to reduced levels of AMH, while the removal of endometriomas improves follicular access and ovarian response to assisted reproductive techniques [48]. Similarly, laparoscopic cystectomy as a treatment for endometriomas diagnosed early in the adolescence remains debatable, since the long-term presence of endometriomas is associated with the appearance of fibrosis in the ovarian cortex and smooth muscle metaplasia with detrimental effects on the ovarian reserve. On the other hand, ovarian tissue loss during laparoscopic cystectomy is inevitable [49]. Therefore, here it is possible that sclerotherapy could provide the solution as the first-choice treatment, though large randomized clinical trials should provide efficient evidence first.

The above results demonstrate the effectiveness of sclerotherapy both in the treatment of pain and infertility related to endometriosis. However, the possible complications of sclerotherapy should be taken into account too. A systematic review and meta-analysis by Cohen et al. showed that abdominal pain is the most common complication found in 1.8-15.3% of the patients, and postoperative fever is present in 5.5%. Alcohol intoxication is found in 3.8%, while intracystic abscess appears in 2% [19].

Our results prove the need of a personalized treatment of endometriosis depending on patients' symptoms and needs. Some studies suggest correlation between intestinal, immune, and neuroendocrine systems in several gynaecological conditions, including endometriosis [20]. Moreover, changes in intestinal microbial composition are found to influence immunosurveillance [50]. Further prospective studies could focus on the use of microbiome to identify the best candidates for each treatment option. Intestinal or reproductive tract microbiota may be associated with fertility outcomes [51]. These findings could be helpful in choosing the best personalized treatment to optimize pregnancy achievement and minimize recurrences and symptoms.

In theory, replacing the abnormal endometrial stromal cells with normal ones could lead to the cure of endometriosis. Abnormal endometrial stromal cells can settle into the pelvic cavity through retrograde flow during menstruation, contributing to the onset and progression of endometriosis. In recent years, pluripotent stem cells have been derived from bone marrow and skin biopsies, which can differentiate into different cell types and be used to treat various diseases [52]. Indeed, differentiation of human stem cells into endometrial stromal fibroblasts has been achieved recently. In this way, fibroblasts could be obtained from the skin of patients with endometriosis, which could then turn into pluripotent stem cells. These can be reprogrammed to create healthy endometrial stromal cells that can replace the abnormal stromal cells of patients with endometriosis, opening new horizons for an induced pluripotent stem-cell based treatment of the disease [53].

Limitations

The main limitations of our review was the small number of patients, especially for laparoscopic sclerotherapy group and studies’ heterogeneity. More prospective studies are necessary to be conducted to provide more data for a validated comparison. Moreover, the follow-up time for the studies was different, with a range from 6 to 53 months, making it hard to extract reliable results.

## Conclusions

Sclerotherapy for ovarian endometriomas is gaining ground against traditional laparoscopic cystectomy. For U/S sclerotherapy, data show a minimum effect on the ovarian reserve, in contrast to laparoscopic methods, making it the ideal choice for patients who have not completed their family planning. At the same time, recurrence rate and clinical pregnancy rate in U/S sclerotherapy are like those of the laparoscopic cystectomy group, while the method seems to lack regarding the symptoms’ relief.

Laparoscopic sclerotherapy has a similar effect on ovarian reserve compared to laparoscopic cystectomy. On the contrary, data shows that this method achieved the best clinical pregnancy rate, which is one of the main reasons why patients choose sclerotherapy over cystectomy. Moreover, the former seems to have two times smaller recurrence rate of endometriomas. Although there is no available data, we believe that laparoscopic sclerotherapy is more effective in symptoms relief than U/S sclerotherapy, since in this way deep endometriosis can be treated and adhesiolysis can be performed too.

All of the three treatment options of ovarian endometriomas seem to be accepted, each with advantages and disadvantages. The choice should be personalized and based on both patient’s desire and physician’s familiarity. More studies need to be conducted and more data should be collected for all three methods. Only then will the results be validated and superiority of each technique documented.
